# Effect of Nb Microalloying on the Dynamic Recrystallization, Bainitic Microstructure, and Mechanical Properties of Hot-Rolled Bainitic Steels

**DOI:** 10.3390/ma19183982

**Published:** 2026-09-19

**Authors:** Rui Cao, Shangqing Chen, Junheng Gao, Haitao Zhao, Qingxiao Feng, Honghui Wu, Chaolei Zhang, Yuhe Huang, Jun Lu, Shuize Wang, Xinping Mao

**Affiliations:** 1 Institute for Carbon Neutrality, University of Science and Technology Beijing, Beijing 100083, China; d202310777@xs.ustb.edu.cn (R.C.);; 2 Institute for Steel Sustainable Technology, Liaoning Academy of Materials, Shenyang 110004, China; 3 Jiangsu (ShaGang) Iron and Steel Research Institute, Zhangjiagang 215625, China

**Keywords:** strip casting, niobium microalloying, high-strength steel, hot rolling process, austenite recrystallization, bainitic transformation

## Abstract

Controlling prior austenite morphology during single-pass hot rolling is essential for improving the strength–ductility balance of strip casting high-strength steels. In this study, Fe–0.24C–1.5Si–1.8Mn steels with and without 0.03 wt.% Nb were deformed by 50% at 950–1100 °C and subsequently held at 350 °C for 60 min for bainitic transformation. The effects of deformation temperature and Nb addition on austenite dynamic recrystallization, bainitic microstructure, and mechanical properties were investigated. For the Nb-free steel, recrystallization occurred at all these investigated deformation temperatures. The addition of 0.03 wt.% Nb markedly suppressed austenite recrystallization during hot deformation, resulting in the retention of elongated deformed austenite after deformation at 950 °C. Compared with the bainitic microstructure formed from fine recrystallized austenite in the 0Nb steel (without Nb addition), that formed from elongated austenite in the 03Nb steel (with 0.03 wt.% Nb) deformed at 950 °C exhibited a smaller lath width and higher HAGB and dislocation densities. In addition, the volume fraction of retained austenite increased from 4.5% to 9.8%, while its average thickness decreased from 105.6 nm to 49.1 nm. Consequently, under identical deformation conditions (50% reduction at 950 °C), Nb addition increased the yield and tensile strengths from 1064 MPa and 1261 MPa to 1088 MPa and 1323 MPa, respectively, while the total elongation remained nearly unchanged (24.8% vs 24.6%). These findings provide new insights into the optimization of thermomechanical processing for strip casting bainitic steels.

## 1. Introduction

To reduce carbon emissions from the steel industry, the development of short-process steel manufacturing technologies has become an important research direction [1,2,3]. Strip casting produces 1.4–2.1-mm-thick strips by rapidly solidifying molten steel between two counter-rotating rolls at cooling rates of approximately 10^2^–10^3^ °C/s [4,5]. After casting, the strip may be directly cooled or subjected to single-pass hot rolling to obtain a final thickness of 0.9–2.1 mm. Compared with conventional processing routes, strip casting eliminates the reheating of the cast strip and substantially simplifies subsequent rolling operations, thereby offering significant advantages in reducing energy consumption and CO_2_ emissions [6]. Accordingly, strip casting requires only approximately 16% and 32% of the energy consumed by conventional steel production and compact strip production, respectively [7]. However, after rapid solidification, the single hot-rolling pass renders the refinement of the as-cast austenite structure very challenging. The resulting coarse prior austenite grains readily transform into coarse bainitic and/or martensitic microstructures, which provide high strength but often insufficient ductility.

Considerable effort has therefore been devoted to improving the strength–ductility balance of strip casting high-strength steels [8,9,10,11]. Xiong et al. [8] developed a strip casting TRIP steel consisting of a multiphase microstructure comprising polygonal ferrite, bainite, retained austenite, and martensite through alloy design and a complex two-stage transformation process, which exhibits a tensile strength of 696 MPa and a total elongation of 26%. Xu et al. [10] employed offline aging to increase the yield strength of a strip casting Nb–V microalloyed steel from 698 to 758 MPa, while increasing the total elongation from 7% to 12%. Huang et al. [11] increased the yield strength from 537 to 585 MPa and the total elongation from 10.0% to 19.1% by introducing NbN nanoclusters and precipitates into the single-phase ferritic matrix. These studies have primarily focused on alloy design, coiling-temperature control, and additional offline treatments to optimize microstructure and mechanical properties. Nevertheless, the potential effects of single-pass hot rolling conditions on optimizing prior austenite morphology to mitigate the detrimental effects of coarse prior austenite grains on the final microstructure and mechanical properties have not been systematically investigated.

In conventional processing, hot rolling regulates the final microstructure and mechanical properties primarily by controlling the size and morphology of prior austenite [12,13,14]. Rolling in the recrystallization region promotes the formation of equiaxed austenite grains, whereas rolling below the recrystallization temperature results in grains elongated along the deformation direction, producing deformed austenite grains [15,16,17]. Such unrecrystallized austenite retains high densities of deformation-induced dislocations, subgrain boundaries, and deformation bands until the onset of bainitic transformation [18,19]. Relative to recrystallized austenite, deformed austenite containing abundant defects offers additional nucleation sites for bainite while suppressing bainitic lath growth and coarsening [20,21]. Chen et al. [22] reported that deformed austenite reduced the average width and length of the resulting bainitic laths from approximately 0.724 and 4.398 μm to 0.151 and 0.830 μm, respectively, while increasing the tensile strength and total elongation from approximately 1521 MPa and 14.7% to 1755 MPa and 18.1%, respectively. However, in strip casting, conventional multi-pass hot rolling is replaced by single-pass hot rolling, making it difficult to lower the finish rolling temperature into the non-recrystallization region. Nb is one of the most effective microalloying elements for suppressing austenite recrystallization during hot deformation [23]. Solute Nb slows austenite grain-boundary migration through the solute-drag effect, whereas strain-induced Nb(C,N) precipitates exert a strong pinning effect on austenite grain boundaries [24,25]. These effects significantly slow austenite recrystallization and recovery, thereby allowing the deformation-induced defects retained in the deformed austenite during the single-pass hot rolling.

Although the role of Nb in slowing austenite recrystallization is well established, how Nb-induced changes in prior austenite morphology affect subsequent bainitic transformation, microstructural evolution, and mechanical properties requires further clarification under single-pass hot rolling condition. Specifically, it is necessary to determine how Nb-induced changes in austenite recrystallization affect bainitic lath refinement, and the fraction and morphology of retained austenite. In this study, steels with and without 0.03 wt.% Nb were subjected to single-pass hot compression at 950–1100 °C, followed by isothermal holding at 350 °C. The effects of Nb addition and deformation temperature on prior austenite grain size (PAGs) and morphology were investigated. Detailed microstructural characterization and tensile testing were then performed on the two steels deformed at 950 °C to establish the relationships between prior austenite morphology, the resulting microstructure, and mechanical properties. This study aims to provide guidance for optimizing thermomechanical processing conditions relevant to strip casting and improving the strength–ductility balance of high-strength steels through control of prior austenite morphology.

## 2. Materials and Methods

### 2.1. Materials Preparation

The investigated steels contained 0 and 0.03 wt.% Nb and were designated as 0Nb and 03Nb, respectively. A Nb content of 0.03 wt.% was selected to suppress austenite recrystallization while limiting coarse NbC formation and alloying costs [26,27,28]. The chemical compositions of the two steels were measured using an ARL iSpark optical emission (Thermo Fisher Scientific (Ecublens) SARL, Ecublens, Switzerland) spectrometer and are listed in Table 1. The steels were produced from high-purity elements by arc melting under a low-pressure, high-purity argon atmosphere. To ensure compositional homogeneity, each ingot was remelted at least five times before being cast into a water-cooled copper mould with dimensions of 15 × 15 × 90 mm. Figure S1 shows the dendritic morphology of the sample produced by copper-mould suction casting, with an average secondary dendrite arm spacing of 22.9 μm. The cooling rate was estimated using the empirical relationship $v={\left(148,/,\lambda_{2}\right)}^{1/0.38}$, where *v* is the cooling rate in °C/s and *λ*_2_ is the secondary dendrite arm spacing in μm. The cooling rate was estimated to be 136 °C/s, within the sub-rapid solidification range typical of strip casting (10^2^–10^3^ °C/s). This result indicates that copper-mould suction casting can approximate the sub-rapid solidification conditions of strip casting to some extent. However, differences in cooling direction, interfacial pressure, and surface constraints between copper-casting mould and industrial twin-roll strip casting mean that the two processes cannot be considered fully equivalent.

**Figure 1:**
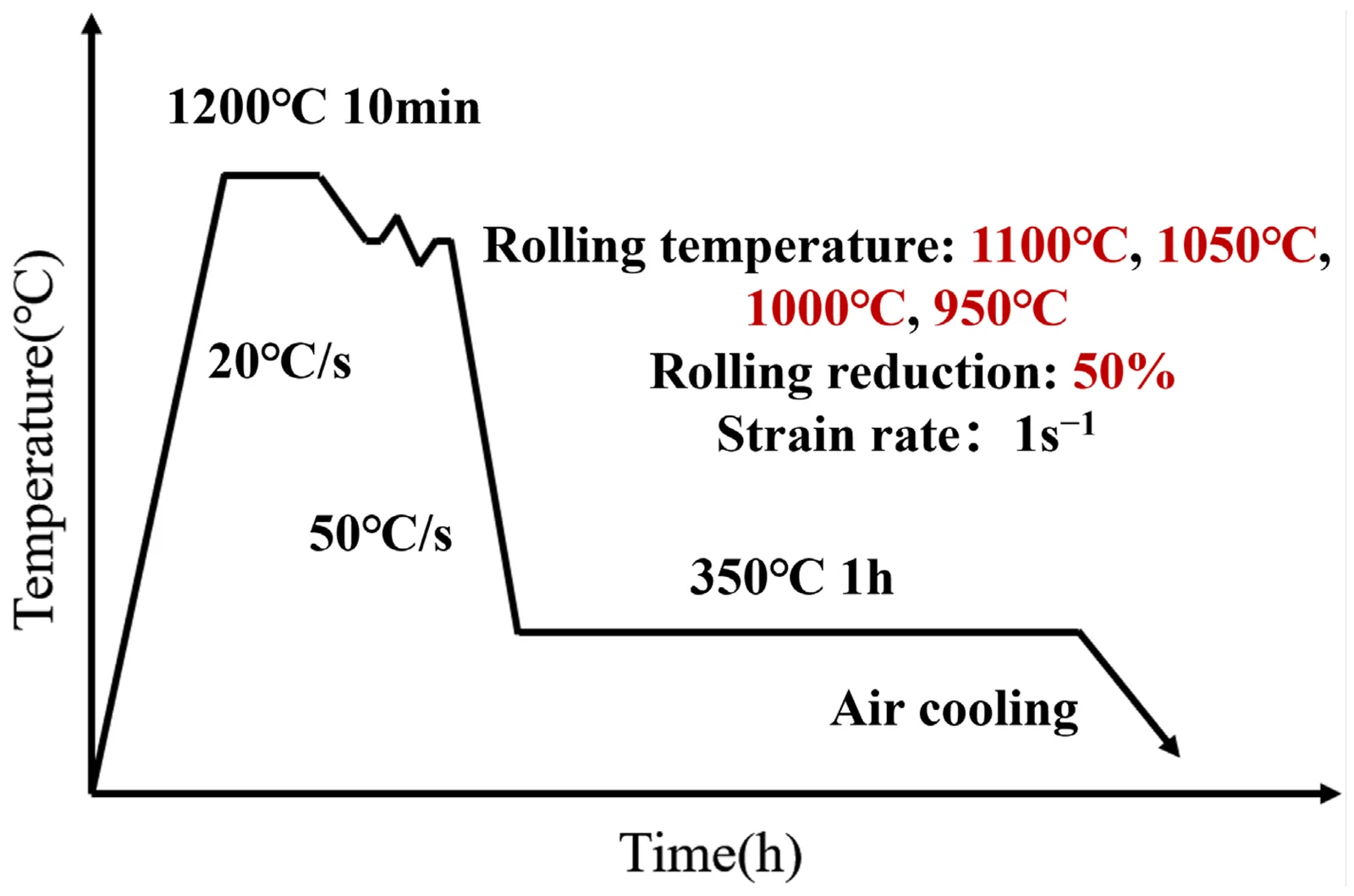
Schematic illustration of the heat-treatment procedures.

**Figure 2:**
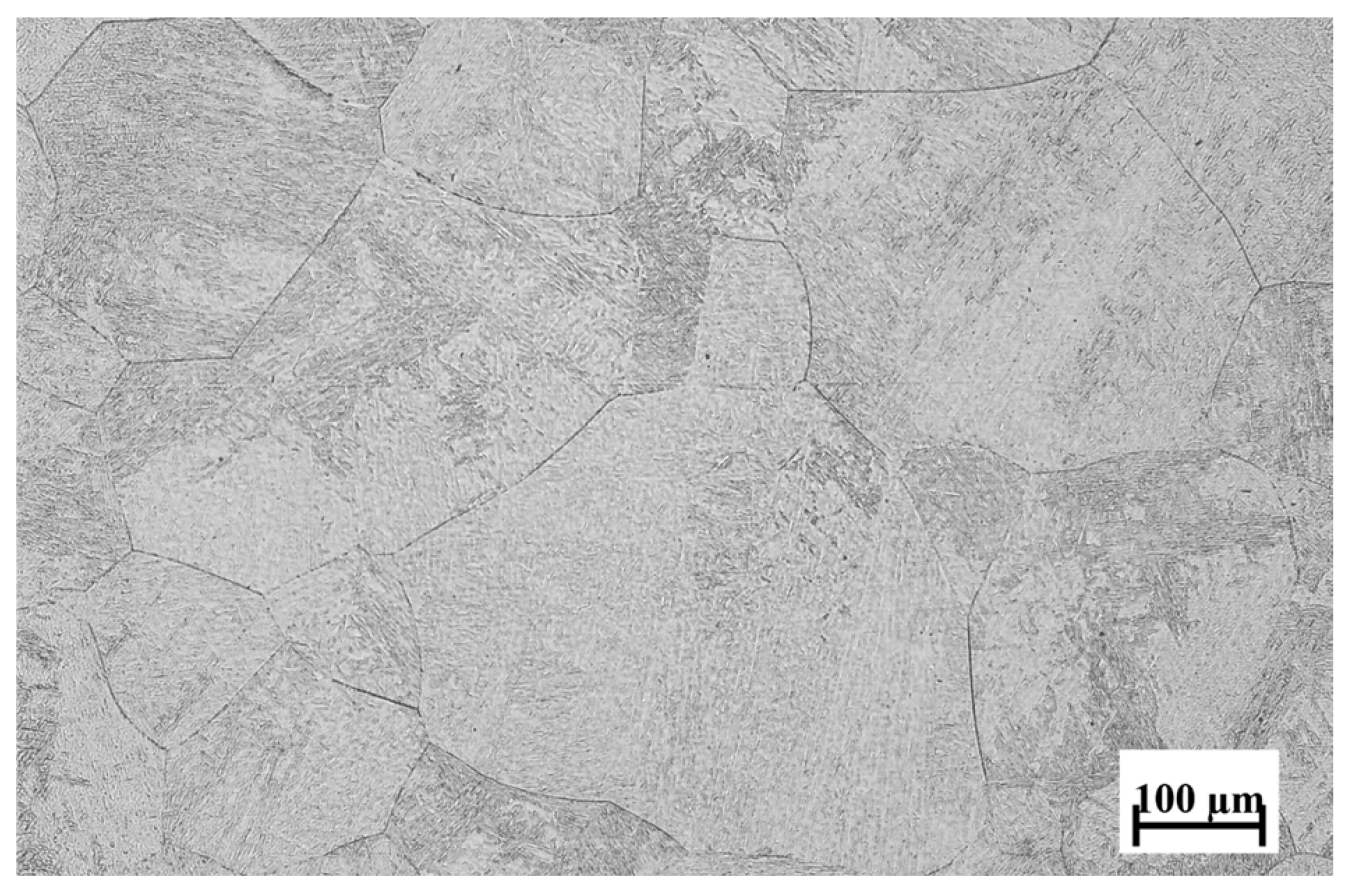
Prior austenite grain morphology of the undeformed reference sample after annealing at 1200 °C for 10 min.

Cylindrical samples measuring 8 mm in diameter and 12 mm in height were machined for hot compression tests using a Gleeble 3800 thermomechanical simulator (Dynamic Systems Inc. (DSI), Poestenkill, NY, USA). To accurately simulate the thermal processing parameters inherent to strip casting, the samples were austenitized at 1200 °C for 10 min, then the samples were cooled to deformation temperatures of 950 °C, 1000 °C, 1050 °C, and 1100 °C at 10 °C/s. A reduction of 50% was applied at a strain rate of 1 s^−1^. After deformation, the samples were cooled to 350 °C at 50 °C/s, held for 60 min to simulate coiling, and subsequently air-cooled to room temperature, as illustrated in Figure 1. In addition, to provide a reference for evaluating austenite recrystallization during single-pass hot rolling, an undeformed reference sample was cooled directly to room temperature at 50 °C/s after annealing at 1200 °C for 10 min. Its apparent PAGs was 181.5 μm, as shown in Figure 2.

Thermodynamic calculations were performed using Thermo-Calc 2025a software with the TCFE13 thermodynamic database (v13.1). The thermodynamic calculation results for the 03Nb steel are shown in Figure S2. The calculated complete dissolution temperature of NbC was approximately 1180 °C, indicating that NbC was fully dissolved at 1200 °C.

### 2.2. Microstructural and Mechanical Property Characterizations

The phase constitution and microstructures of the 0Nb and 03Nb steels were characterized by X-ray diffraction (XRD), scanning electron microscopy (SEM), electron backscatter diffraction (EBSD), and transmission electron microscopy (TEM). Samples for SEM observation were mechanically ground, polished, and etched with 4% Nital. SEM characterization was performed using a Tescan Mira microscope (Tescan, Brno, Czech Republic) operated at 20 kV with a working distance of 15 mm. For EBSD analysis, samples were sectioned from the gauge regions, mechanically polished, and then electropolished at 15 V for 15 s in an ethanol solution containing 6 vol.% perchloric acid. EBSD maps were acquired using an Oxford Instruments Symmetry S2 detector (Oxford Instruments plc, High Wycombe, UK) at an accelerating voltage of 25 kV and a step size of 0.1 μm, and the data were analyzed using AztecCrystal software (v4.0). TEM samples were also taken from the gauge regions, ground to a thickness of approximately 55 μm, punched into 3-mm-diameter discs, and twin-jet electropolished using a Tenupol-5 system (Struers) (Struers ApS, Ballerup, Denmark) in an electrolyte containing 6 vol.% perchloric acid and 94 vol.% ethanol at −30 °C. TEM observations were conducted using a JEOL 2100F microscope (JEOL Ltd., Akishima, Tokyo, Japan) operated at 200 kV.

The apparent PAGs was measured on two-dimensional sections using the line-intercept method in accordance with ISO 643 [29]. For each condition, test lines covering more than 200 prior austenite grains were analyzed using Nano Measurer (v1.2) software.

XRD measurements were conducted using a Bruker D8 Advance diffractometer (Bruker AXS GmbH, Karlsruhe, Germany) with Cu Kα radiation for phase identification and dislocation density estimation. The diffraction patterns were collected with a step size of 0.01° and a scanning rate of 2° min^−1^ at a tube voltage of 40 kV and a current of 150 mA. An Al_2_O_3_ standard sample was measured under identical conditions to determine the instrumental broadening, which was subsequently removed from the measured peak broadening to obtain the sample-induced broadening. The resulting XRD patterns were analyzed using Jade 6.0 software. The volume fraction of retained austenite (RA) was calculated using Equation (1) based on the integrated intensities of the (200)γ, (220)γ, and (311)γ austenite reflections and the (200)α and (211)α ferrite reflections [30].

(1) $$V_{\gamma }=\frac{\left(1/n\right)\Sigma_{j=1}^{n}I_{\gamma }^{\dot{J}}∕R_{\gamma }^{j}}{\left(1/n\right)\Sigma_{j=1}^{n}\left(I_{\gamma }^{j}∕R_{\gamma }^{j}\right)+\left(1/n\right)\Sigma_{j=1}^{n}\left(I_{\alpha }^{j}∕R_{\alpha }^{\dot{J}}\right)}$$

where *n* is the number of diffraction peaks included in the calculation, *I* is the integrated intensity of the diffraction peak, and R is the corresponding material scattering factor.

The dislocation densities (*ρ*) of the two steels were estimated from the XRD peak positions and full widths at half maximum (FWHMs) using the modified Williamson–Hall (MWH) method [31,32]. The XRD patterns were recorded over a 2θ range of 40–120°, and the (110), (200), (211), (220), and (310) reflections of the α phase were used in the analysis. The dislocation density was determined using the following equations [33]:

(2) $$\Delta K=0.9/D+{\left(\pi M^{2}b^{2}/2\right)}^{1/2}\rho^{1/2}\left(KC^{1/2}\right)$$

(3) $$\Delta K=2cos\;\delta [\Delta \left(2\theta \right)]/\lambda$$

(4) K=2sin θ/λ

(5) $$C=1-\frac{q\left(h^{2}k^{2}+k^{2}l^{2}+l^{2}h^{2}\right)}{{\left(h^{2}+k^{2}+l^{2}\right)}^{2}}$$

(6) $$\rho =2\beta^{2}/\pi M^{2}b^{2}$$

where *δ*, Δ(2*θ*), and *λ* denote the diffraction angle, full width at half maximum (FWHM), and X-ray wavelength (*λ* = 0.154 nm for Cu Kα radiation), respectively. *D*, *ρ*, and *b* represent the average crystallite size, dislocation density, and magnitude of the Burgers vector (*b* = 0.248 nm), respectively. *M* is a dimensionless parameter related to the effective outer cut-off radius and density of dislocations. For steels, *M* generally ranges from 1 to 2 [34,35], and a value of 2 was adopted in this study [36]. The parameters *h*, *k*, and *l* are the Miller indices of each reflection, *q* characterizes the dislocation type, and *β* is the slope obtained by plotting Δ*K* and *KC*^1/2^.

Flat tensile samples had an overall length of 10 mm, a gauge length of 2 mm, a thickness of 1 mm, and a width of 1.25 mm. Room-temperature tensile tests were conducted using an MTS E40 universal testing machine (MTS Systems Corporation, Eden Prairie, MN, USA) at a strain rate of 1 × 10^−3^ s^−1^. At least three samples were tested under each condition.

## 3. Results and Discussion

### 3.1. Effects of Hot Rolling Temperature and Nb Microalloying on the Dynamic Recrystallization Behaviors of Strip Casting Bainitic Steels

**Figure 3:**
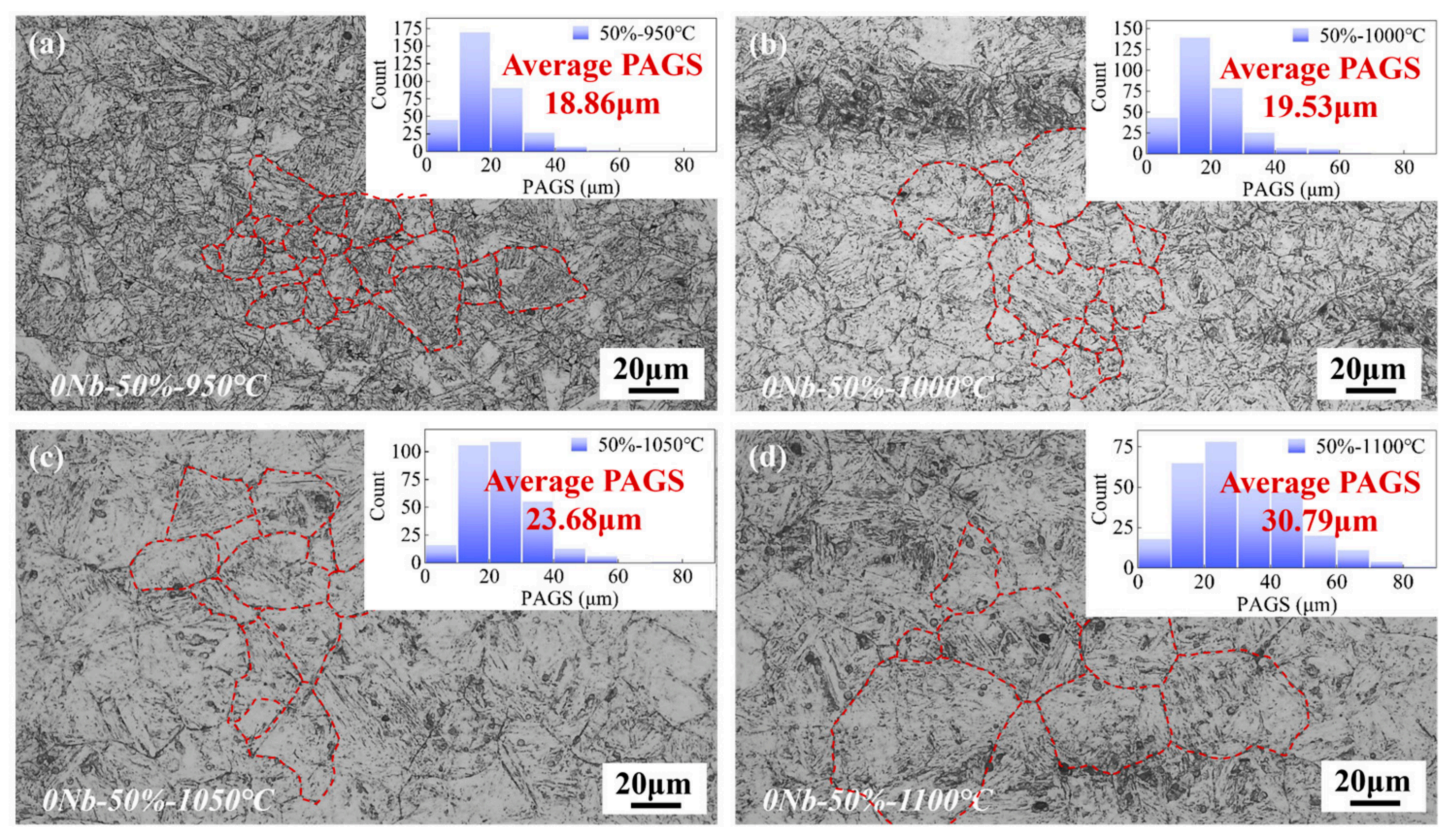
Prior austenite morphologies and corresponding grain-size distributions (insets) of the 0Nb steel after 50% deformation at (**a**) 950 °C, (**b**) 1000 °C, (**c**) 1050 °C, and (**d**) 1100 °C. (Red solid lines indicate the prior austenite grain boundaries).

The prior austenite morphologies and corresponding grain-size distributions of the 0Nb steel after 50% deformation at different temperatures are presented in Figure 3. The reported morphologies and grain-size distributions represent two-dimensional grain sections in the observation plane. As shown in Figure 3a–d, nearly equiaxed prior austenite grains are observed for all four conditions, without pronounced elongation along the deformation direction being observed, indicating that extensive austenite recrystallization occurred in the Nb-free steel over the four investigated temperatures. The average PAGs increased progressively with increasing deformation temperature, reaching 18.86 μm, 19.53 μm, 23.68 μm and 30.79 μm after 50% deformation at 950 °C, 1000 °C, 1050 °C and 1100 °C, respectively. The recrystallization grain size *d_RX_* can be expressed as Equations (7) and (8) [37,38]:

(7) $$d_{Rx}=BZ^{-P}$$

(8) $$Z=\varepsilon \exp\left(\frac{Q}{RT}\right)$$

where *B* and *P* are constants, *Z* is the Zener–Hollomon parameter, *Q* is the hot deformation activation energy, *R* is the universal gas constant, *T* is the deformation temperature, and *ε* is a strain rate of 1 s^−1^. According to Equations (7) and (8), the higher Zener–Hollomon parameter at lower deformation temperatures suppressed austenite grain-boundary migration and dynamic recovery. The resulting retention of stored deformation energy and high densities of dislocations and subgrain boundaries at lower deformation temperature favored the nucleation of recrystallized austenite grains [39,40], while the limited boundary mobility restricted their subsequent growth. Consequently, the austenite retained a fine-grained morphology after deformation at 950 °C. By contrast, as the deformation temperature increased, enhanced dynamic recovery of austenite reduced the density of potential nucleation sites for recrystallization. Meanwhile, accelerated atomic diffusion and greater grain-boundary mobility promoted the subsequent growth of recrystallized grains, resulting in a larger recrystallized austenite grain size [41].

**Figure 4:**
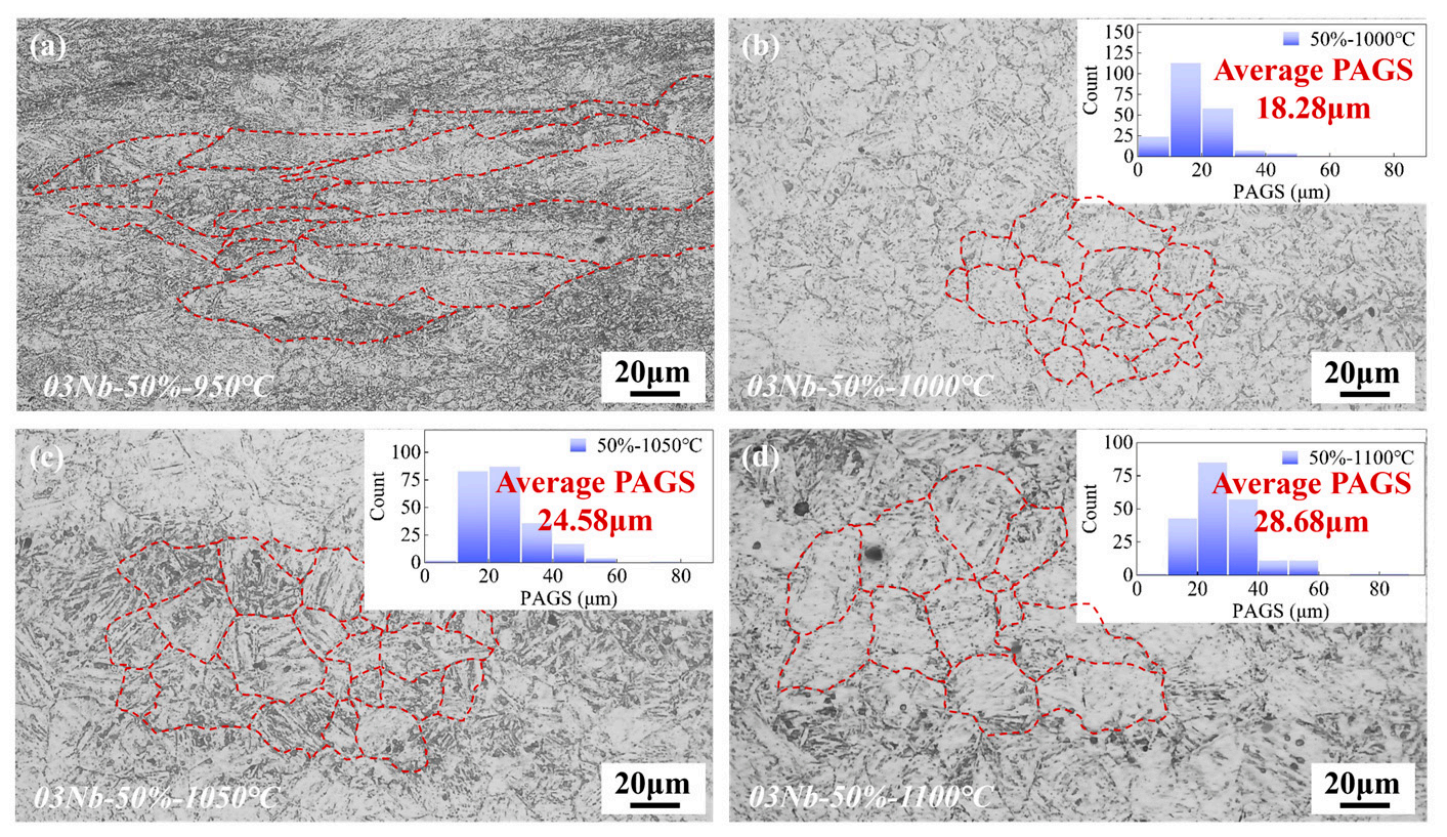
Prior austenite morphologies and corresponding grain-size distributions (insets) of the 03Nb steel after 50% deformation at (**a**) 950 °C, (**b**) 1000 °C, (**c**) 1050 °C, and (**d**) 1100 °C. (Red solid lines indicate the prior austenite grain boundaries).

The prior austenite morphologies and corresponding grain-size distributions of the 03Nb steel after 50% deformation at different temperatures are presented in Figure 4. At 950 °C, the prior austenite grains remained markedly elongated along the deformation direction, indicating that austenite recrystallization was strongly suppressed. At deformation temperatures of 1000 °C and above, extensive austenite recrystallization produced an equiaxed grain structure, as shown in Figure 4b–d. The PAGs increased progressively with increasing deformation temperature, and the average grain sizes after deformation at 1000 °C, 1050 °C and 1100 °C were 18.28 μm, 24.58 μm and 28.68 μm, respectively.

The formation of viable recrystallization nuclei generally requires the local rearrangement of deformation-induced dislocations and the progressive development of high-angle boundaries [42,43]. The solute-drag effect of Nb markedly slows dislocation rearrangement as well as boundary migration [26], thereby necessitating more favorable deformation conditions for the formation of viable recrystallization nuclei (e.g., a higher deformation temperature or a larger deformation strain) [44]. Therefore, the addition of 0.03 wt.% Nb markedly suppressed austenite recrystallization during deformation at 950 °C, retaining a large fraction of elongated deformed austenite grains. However, when the deformation temperature was increased to 1000 °C or above, enhanced thermal activation facilitated dislocation motion and boundary migration, enabling recrystallized austenite grains to nucleate and grow. Moreover, progressive grain coarsening occurred with increasing deformation temperature. As a result, the average PAGs increased with deformation temperature, as shown in Figure 4b–d.

In addition, as shown in Figure 3b–d, the average PAGs of the 0Nb steel after deformation at 1000 °C, 1050 °C, and 1100 °C were 19.53 μm, 23.68 μm, and 30.79 μm, respectively, which were comparable to those of the 03Nb steel under the corresponding deformation conditions. This indicates that once austenite recrystallization occurred during single-pass hot rolling in strip casting, the addition of 0.03 wt.% Nb did not significantly inhibit the subsequent growth of the recrystallized grains. Owing to the sub-rapid solidification and low-strain single-pass rolling characteristics of strip casting, Nb predominantly remained in solid solution rather than forming abundant NbC precipitates (as will be confirmed by the TEM results). In the absence of a substantial Zener pinning effect of NbC precipitates, the solute-drag effect exerted by solute Nb alone was insufficient to restrict the migration of recrystallized austenite grain boundaries at 1000–1100 °C [25,45]. Consequently, the recrystallized austenite grain sizes of the 0Nb and 03Nb steels were comparable in each deformation temperature.

### 3.2. Effect of Prior Austenite Morphology on the Microstructure of Strip Casting Bainitic Steel

To further investigate the effects of prior austenite morphology on the subsequent microstructure and mechanical properties, detailed microstructural characterization was performed on the 0Nb and 03Nb samples after 50% deformation at 950 °C, isothermal holding at 350 °C for 60 min, and subsequent air cooling to room temperature.

**Figure 5:**
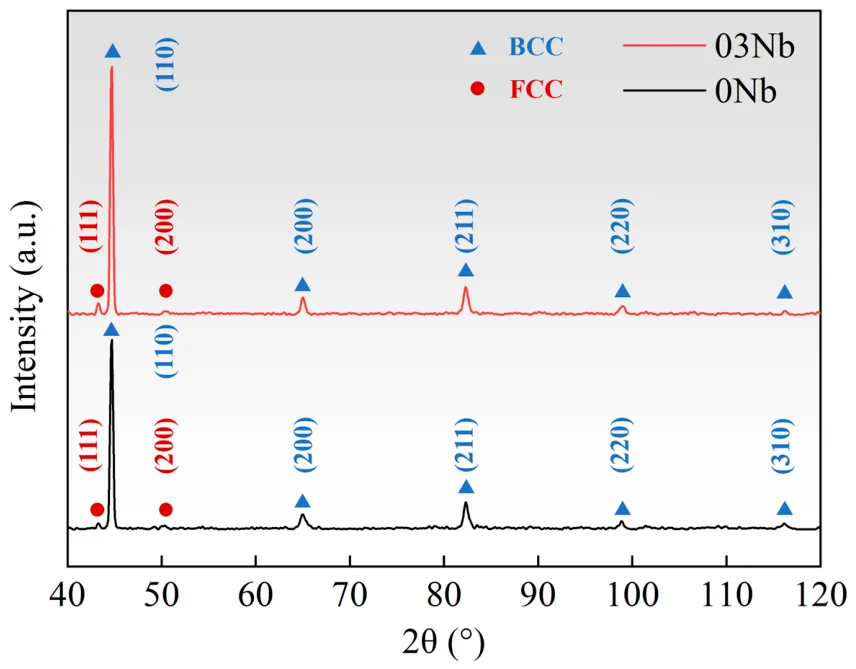
X-ray diffraction patterns of the 0Nb and 03Nb steels deformed at 950 °C.

The XRD patterns of the two steels are presented in Figure 5. Both patterns contain BCC and FCC diffraction peaks. The BCC reflections are mainly associated with bainitic, while the FCC reflections confirm the presence of RA in both steels. The relative intensities of the FCC peaks in the 03Nb steel were higher than those in the 0Nb steel. According to Equation (1), the RA volume fractions of the 0Nb and 03Nb steels were calculated to be 4.5% and 9.8%, respectively. The dislocation densities calculated using Equations (2)–(6) were 9.8 × 10^13^ m^−2^ and 1.80 × 10^14^ m^−2^ for the 0Nb and 03Nb steels, respectively.

**Figure 6:**
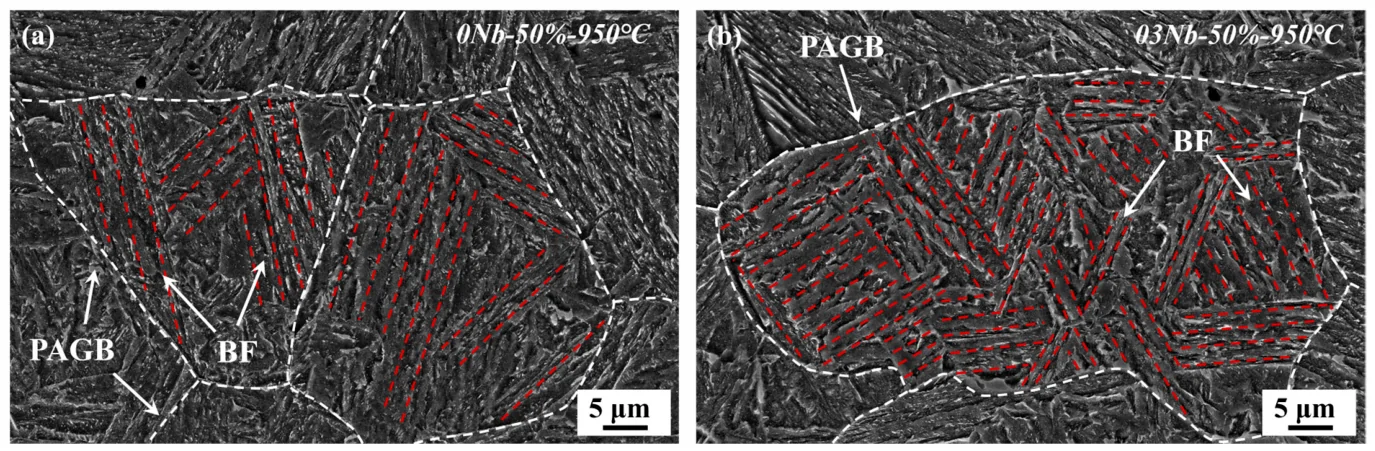
SEM microstructures of the (**a**) 0Nb and (**b**) 03Nb steels deformed at 950 °C.

The SEM microstructures of the 0Nb and 03Nb steels after 50% deformation at 950 °C, isothermal holding at 350 °C for 60 min, and subsequent air cooling to room temperature are presented in Figure 6. The white dashed lines delineate the prior austenite grain boundaries, while the red dashed lines indicate the orientations of the bainitic lath bundles. As shown in Figure 6a, relatively long and parallel bainitic lath bundles formed within the fine recrystallized austenite grains of the 0Nb steel. By contrast, the deformed austenite in the 03Nb steel produced a finer bainitic microstructure, characterized by shorter lath bundles and a greater number of bundle orientations, as shown in Figure 6b. The deformed austenite in the 03Nb steel retained a high density of deformation-induced dislocations, subgrain boundaries, and deformation bands, which provided additional intragranular nucleation sites for bainitic transformation [46]. The increased nucleation density promoted the formation of more lath bundles with different orientations, thereby promoting impingement between neighboring bainitic lath bundles during growth and restricting their further extension [21]. In addition, the high density of dislocations and deformation substructures retained in the deformed austenite impeded the migration of austenite/bainitic interfaces [47], further restricting bainite growth. These combined effects produced the shorter and finer lath bundles observed in the 03Nb steel.

**Figure 7:**
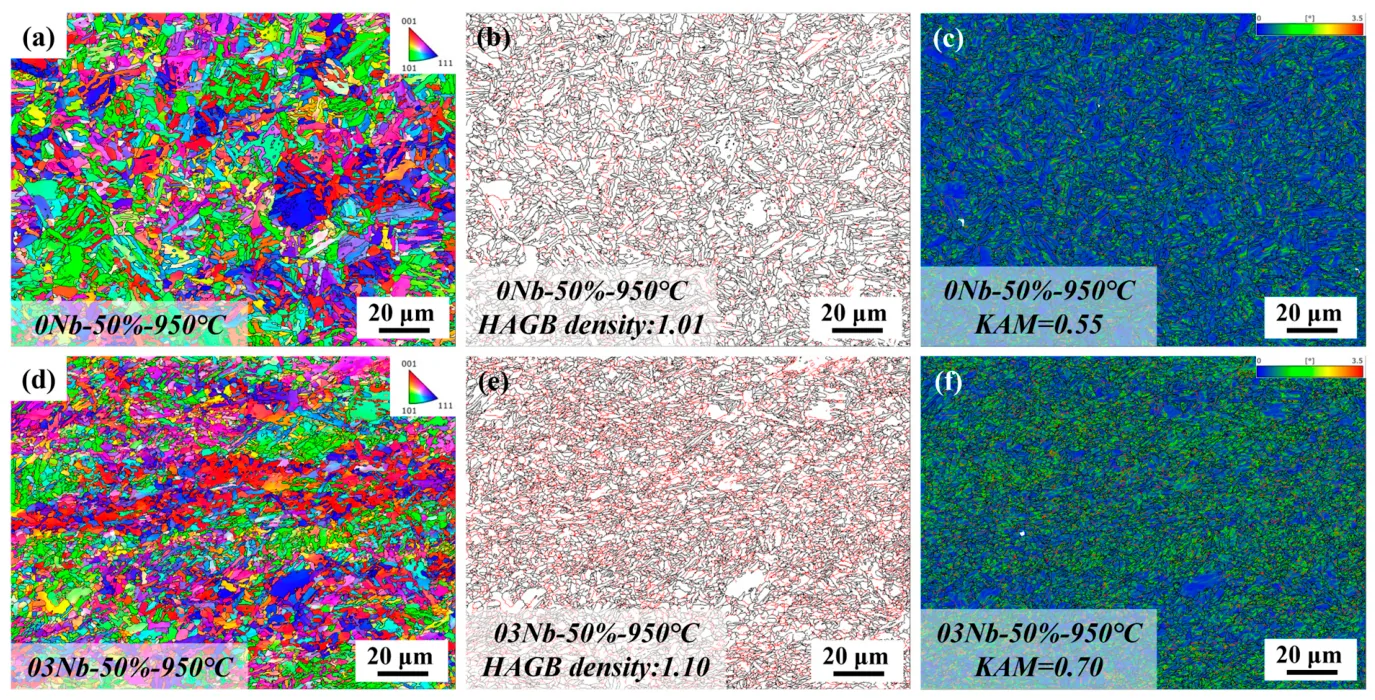
EBSD maps of the (**a**–**c**) 0Nb and (**d**–**f**) 03Nb steels after deformation at 950 °C: (**a**,**d**) inverse pole figure maps; (**b**,**e**) grain-boundary misorientation maps; and (**c**,**f**) kernel average misorientation maps.

To further clarify the influence of prior austenite morphology on the transformed bainitic microstructure, EBSD analysis was performed on the 0Nb and 03Nb steels. Figure 7a and Figure 7d show the inverse pole figure (IPF) maps of the two steels, respectively. The elongated band-like arrangement of the crystallographic units in the 03Nb steel clearly reveals the morphological characteristics inherited from the deformed austenite. The grain-boundary misorientation maps in Figure 7b,e distinguish HAGBs (θ ≥ 15°) by black lines and LAGBs (2° ≤ θ < 15°) by red lines. Compared with the bainitic microstructure transformed from the recrystallized austenite in the 0Nb steel, the HAGB density of the 03Nb steel increased from 1.01 μm^−1^ to 1.10 μm^−1^. The shorter lath bundles and increased number of orientations subdivided the microstructure into more crystallographically distinct bainitic units, thereby increasing the HAGB density. Owing to their large misorientation, HAGBs effectively impede dislocation glide [48]. The resulting dislocation pile-ups increase the stress required for slip transfer or the activation of dislocation sources in adjacent grains, enabling plastic deformation to continue under a higher applied load [49]. Figure 7c and Figure 7f show the kernel average misorientation (KAM) maps of the 0Nb and 03Nb steels, respectively. The average KAM value increased from 0.55° in the 0Nb steel to 0.70° in the 03Nb steel, indicating greater local lattice distortion and a higher density of geometrically necessary dislocations in the bainitic microstructure transformed from the elongated prior austenite. This result is consistent with the higher dislocation density of the 03Nb steel determined by XRD analysis. As a microstructure characterized by a displacive phase transformation, bainite can retain the dislocations introduced during prior hot rolling [47]. Compared with the recrystallized austenite in the 0Nb steel, the deformed austenite in the 03Nb steel retained a higher density of dislocations. Consequently, the bainitic microstructure formed in the 03Nb steel exhibited a higher dislocation density. Moreover, because the growth of bainitic-ferrite lath bundles was restricted during the transformation of the 03Nb steel, a higher density of dislocations was generated at the bainitic-ferrite/austenite interfaces to accommodate the transformation strain [50,51].

**Figure 8:**
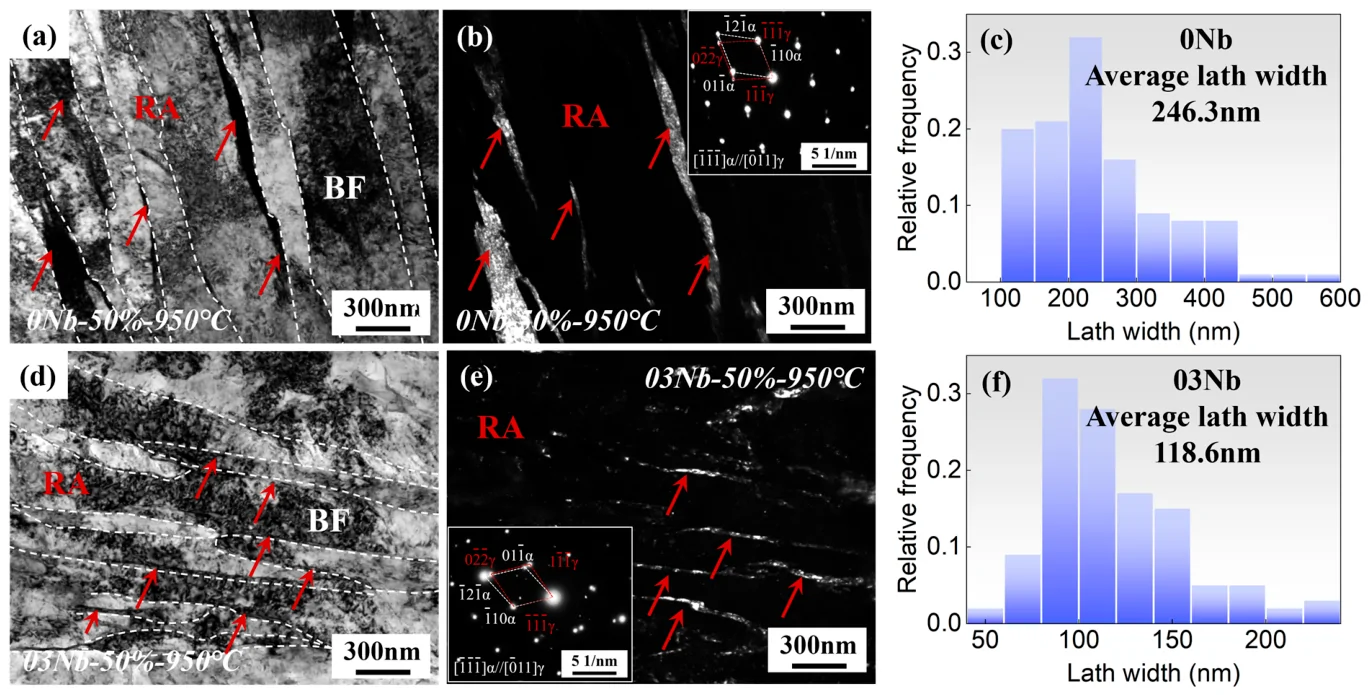
TEM characterization of the 0Nb and 03Nb steels deformed at 950 °C: (**a**,**d**) bright-field images, (**b**,**e**) corresponding dark-field images with selected-area electron diffraction patterns in the insets, and (**c**,**f**) bainitic lath width distributions. (**a**–**c**) 0Nb steel and (**d**–**f**) 03Nb steel.

Bright-field TEM images of the 0Nb and 03Nb steels are presented in Figure 8a and Figure 8d, respectively, with the bainitic lath boundaries delineated by white dashed lines. Compared with the bainite transformed from the recrystallized austenite in the 0Nb steel, the bainitic laths transformed from the deformed austenite in the 03Nb steel were considerably finer. Specifically, the average lath width decreased from 246.3 nm in the 0Nb steel to 118.6 nm in the 03Nb steel, as shown in Figure 8c,f. Figure 8b,e show the corresponding dark-field images. Film-like retained austenite was distributed along the bainitic lath boundaries, with average thicknesses of 105.6 ± 10.9 nm and 49.1 ± 8.6 nm in the 0Nb and 03Nb steels, respectively. As shown in Figure 5 and Figure 8b,e, the RA formed from the deformed austenite exhibited a higher volume fraction (9.8% in the 03Nb steel compared with 4.5% in the 0Nb steel) and a reduced thickness. This is because deformed austenite promoted the high-density nucleation of bainitic and restricted its subsequent growth, resulting in pronounced refinement of the bainitic laths. During the bainitic transformation, the untransformed austenite was repeatedly partitioned by numerous fine bainitic-ferrite laths and eventually developed into thin films between adjacent laths. During subsequent isothermal holding, the short diffusion distances facilitated sufficient carbon enrichment in these fine untransformed austenite regions, thereby enhancing their stability and allowing a larger fraction to be retained at room temperature. The thinner film-like RA in the 03Nb steel possess higher mechanical stability and is less susceptible to premature transformation, thereby providing a more gradual and sustained transformation-induced plasticity (TRIP) effect during plastic deformation [52,53].

As shown in Figure 8d, no NbC precipitates were detected in the 03Nb steel, suggesting that Nb remained predominantly in solid solution in the matrix under the present processing conditions. During single-pass hot rolling, a 50% reduction corresponds to a true strain of 0.693. At a strain rate of 1 s^−1^, the deformation time was therefore only approximately 0.693 s, which was insufficient for the nucleation and growth of strain-induced NbC precipitates. After deformation, the samples were immediately cooled to 350 °C at 50 °C s^−1^ and held for 60 min. During the subsequent isothermal holding at 350 °C, carbon atoms remained sufficiently mobile for local redistribution, whereas the diffusion of substitutional Nb was kinetically negligible [54,55]. Therefore, NbC was not expected to precipitate during the isothermal holding at 350 °C either.

### 3.3. Effect of Microstructure on the Mechanical Properties of Strip Casting Bainitic Steels

**Figure 9:**
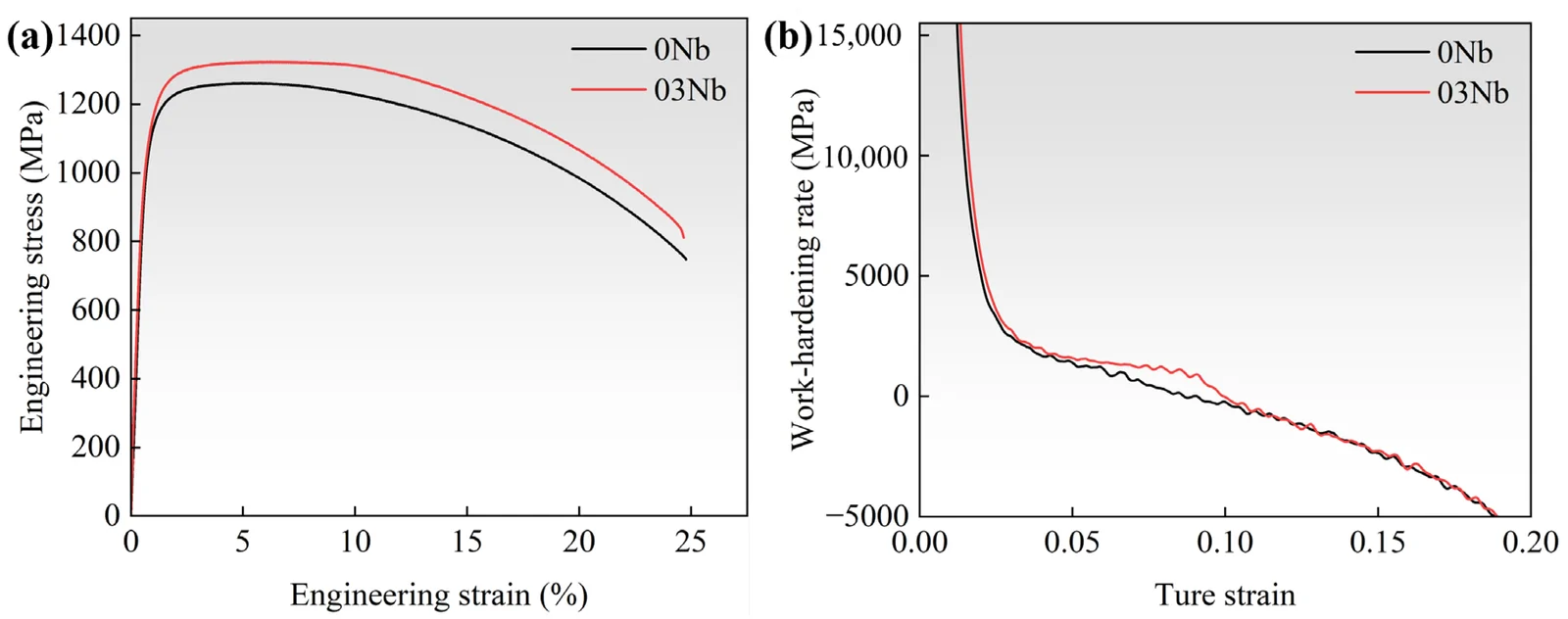
Tensile properties of the 0Nb and 03Nb steels deformed at 950 °C: (**a**) engineering stress–strain curves and (**b**) work-hardening-rate curves.

The engineering stress–strain curves of the 0Nb and 03Nb steels are presented in Figure 9a, while the curves obtained from all replicate tensile tests are provided in Figure S3. The 0Nb steel exhibited a yield strength of 1064 ± 10.4 MPa, an ultimate tensile strength of 1261 ± 13.1 MPa, and a total elongation of 24.8 ± 0.2%. After the addition of 0.03 wt.% Nb, the yield strength and ultimate tensile strength increased to 1088 ± 9.8 MPa and 1323 ± 18.6MPa, respectively, while the total elongation remained nearly unchanged at 24.6 ± 0.3%. The higher strength of the 03Nb steel resulted from the refinement of the bainitic laths and the increased HAGB density, which increased the resistance to dislocation motion. Meanwhile, its higher initial dislocation density provided an additional dislocation strengthening effect.

As shown in Figure 9b, the work-hardening rates of both steels decreased continuously after yielding. However, the work-hardening rate of the 03Nb steel decreased more slowly than that of the 0Nb steel within the true-strain range of 0.05–0.10. Compared with the 0Nb steel, the RA in the 03Nb steel exhibited a higher volume fraction and a smaller average thickness, thereby providing a more sustained TRIP effect and thus maintaining a higher work-hardening rate during plastic deformation [56]. Moreover, bainitic refinement and the increased HAGB density promoted dislocation accumulation and storage at interfaces, further contributing to the higher work-hardening rate [57]. The diverse crystallographic orientations of the bainitic units facilitated the activation of multiple slip systems, thereby improving strain compatibility, reducing local stress concentrations at boundaries, and promoting dislocation interactions and multiplication within grain interiors [58,59]. Consequently, despite its higher initial dislocation density and yield strength, the 03Nb steel retained a total elongation comparable to that of the 0Nb steel owing to its sustained TRIP effect and greater capacity for dislocation multiplication and storage.

## 4. Conclusions

In this study, the effects of deformation temperature and Nb addition on prior austenite evolution, bainitic microstructure, and the mechanical properties of newly developed strip-casting bainitic high-strength steels were systematically investigated. The principal conclusions are summarized as follows:

(1) Nb addition suppressed austenite recrystallization at 950 °C, retaining elongated deformed austenite in the 03Nb steel, whereas the 0Nb steel exhibited a predominantly equiaxed recrystallized structure. At 1000–1100 °C, both steels exhibited recrystallized austenite with comparable measured grain sizes.
(2) In the 03Nb steel, austenite deformation substructures were retained after deformation at 950 °C, which promoted intragranular bainite nucleation and restricted growth during holding at 350 °C, causing the refinement of the bainitic microstructure. Compared with the 0Nb steel, the HAGB density increased from 1.01 to 1.10 μm^−1^, and the bainitic lath width decreased from 246.3 to 118.6 nm. The RA fraction increased from 4.5% to 9.8%, while its average film thickness decreased from 105.6 to 49.1 nm.
(3) Compared with the 0Nb steel, the tensile strength and the yield strength of the 03Nb steel increased from 1261 MPa and 1064 MPa to 1323 MPa and 1088 MPa, corresponding to increases of 62 and 24 MPa, respectively. The higher fraction and smaller film thickness of RA provided a more sustained TRIP effect, while the refined bainitic microstructure enhanced dislocation multiplication and storage. Consequently, the 03Nb steel maintained a total elongation of 24.6%, comparable to the 24.8% of the 0Nb steel.

## Figures and Tables

**Table 1:** Chemical compositions of the 0Nb and 03Nb steels measured by optical emission spectroscopy (wt.%).

Steel	C	Si	Mn	Nb
0Nb	0.24	1.49	1.75	-
03Nb	0.24	1.48	1.78	0.03

## Data Availability

The original contributions presented in this study are included in the article/Supplementary Materials. Further inquiries can be directed to the corresponding author.

## Supplementary Materials

The following supporting information can be downloaded at https://www.mdpi.com/article/10.3390/ma19183982/s1, Figure S1. Dendritic morphology of the sample produced by copper-mould suction casting; Figure S2. Thermodynamic calculation results for the 03Nb steel; Figure S3. Engineering stress–strain curves of all tensile samples of the 0Nb and 03Nb steels.
