# Supplementary material for: Effect of Nb Microalloying on the Dynamic Recrystallization, Bainitic Microstructure, and Mechanical Properties of Hot-Rolled Bainitic Steels
Source: Materials (Basel). 2026 Sep 19;19(18):3982. doi: 10.3390/ma19183982 (PMC13608715; doi:10.3390/ma19183982)
Supplement: Supplementary file 1 [file materials-19-03982-s001.zip › materials-4532619-supplementary.pdf]

Effect of Nb Microalloying on the Dynamic Recrystallization, Bainitic Microstructure, and Mechanical Properties of Hot-Rolled Bainitic Steels

Rui Cao [1], Shangqing Chen [1], Junheng Gao [1,2,*] , Haitao Zhao [1,2], Qingxiao Feng [3], Honghui Wu [1,2], Chaolei Zhang [1,2], Yuhe Huang [1,2], Jun Lu [1,2], Shuize Wang [1,2] and Xinping Mao [1,2]

1 Institute for Carbon Neutrality, University of Science and Technology Beijing, Beijing 100083, China; d202310777@xs.ustb.edu.cn (R.C.)

2 Institute for Steel Sustainable Technology, Liaoning Academy of Materials, Shenyang 110004, China

3 Jiangsu (ShaGang) Iron and Steel Research Institute, Zhangjiagang 215625, China

* Correspondence: junhenggao@ustb.edu.cn; Tel.: +86-010-6233-3990

1. Prior austenite grain morphology of the as-cast specimen

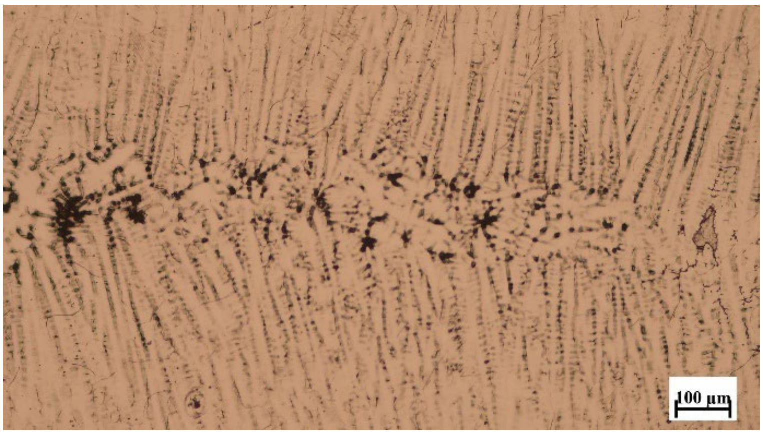

Fig.S1. Dendritic morphology of the sample produced by copper-mould suction casting.

2. Thermodynamic calculation results for the 03Nb steel

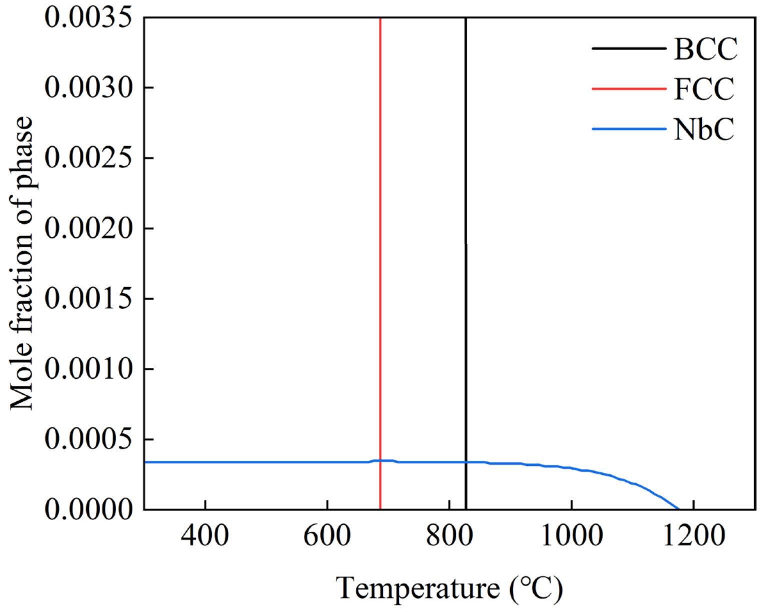

Fig. S2. Thermodynamic calculation results for the 03Nb steel

3. Mechanical properties

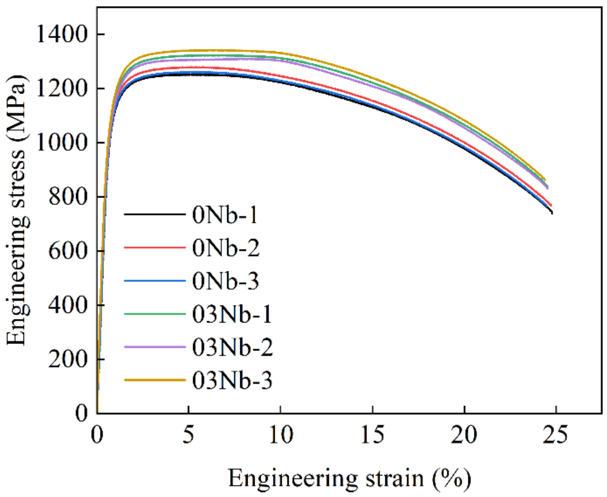

Fig.S3. Engineering stress–strain curves of all tensile samples of the 0Nb and 03Nb steels.
